# Immunological Characterization of Whole Tumour Lysate-Loaded Dendritic Cells for Cancer Immunotherapy

**DOI:** 10.1371/journal.pone.0146622

**Published:** 2016-01-21

**Authors:** Veronica Rainone, Cristina Martelli, Luisa Ottobrini, Mara Biasin, Manuela Borelli, Giovanni Lucignani, Daria Trabattoni, Mario Clerici

**Affiliations:** 1 Department of Biomedical and Clinical Sciences “L. Sacco”, Chair of Immunology, University of Milan, Milan, Italy; 2 Department of Pathophysiology and Transplantation, University of Milan, Segrate, Milan, Italy; 3 Centre of Molecular and Cellular Imaging—IMAGO, University of Milan, Milan, Italy; 4 Institute for Molecular Bioimaging and Physiology (IBFM), National Research Council (CNR), Segrate, Milan, Italy; 5 Departments of Health Sciences, University of Milan, Milan, Italy; 6 Department of Diagnostic Services, Unit of Nuclear Medicine, San Paolo Hospital, Milan, Italy; 7 Don C. Gnocchi Foundation IRCCS, Milan, Italy; Baylor College of Medicine, UNITED STATES

## Abstract

**Introduction:**

Dendritic cells play a key role as initiators of T-cell responses, and even if tumour antigen-loaded dendritic cells can induce anti-tumour responses, their efficacy has been questioned, suggesting a need to enhance immunization strategies.

**Matherials & Methods:**

We focused on the characterization of bone marrow-derived dendritic cells pulsed with whole tumour lysate (TAA-DC), as a source of known and unknown antigens, in a mouse model of breast cancer (MMTV-*Ras*). Dendritic cells were evaluated for antigen uptake and for the expression of MHC class I/II and costimulatory molecules and markers associated with maturation.

**Results:**

Results showed that antigen-loaded dendritic cells are characterized by a phenotypically semi-mature/mature profile and by the upregulation of genes involved in antigen presentation and T-cell priming. Activated dendritic cells stimulated T-cell proliferation and induced the production of high concentrations of IL-12p70 and IFN-γ but only low levels of IL-10, indicating their ability to elicit a T_H_1-immune response. Furthermore, administration of Antigen loaded-Dendritic Cells in MMTV-*Ras* mice evoked a strong anti-tumour response *in vivo* as demonstrated by a general activation of immunocompetent cells and the release of T_H_1 cytokines.

**Conclusion:**

Data herein could be useful in the design of antitumoral DC-based therapies, showing a specific activation of immune system against breast cancer.

## Introduction

Cancer immunotherapy aims at the optimal elicitation of tumour-specific immune responses with the ultimate goal of destroying tumour cells and inducing long lasting immunity that will prevent disease relapse [1]. Induction of effective tumour immunity is a complex process that includes the appropriate presentation of tumour-associated antigens (TAA), the selection and activation of TAA-specific T-cells and, lastly, homing of TAA-specific T-cells to the tumour site and the elimination of malignant cells expressing the TAA [2,3,4]. Escape from immune surveillance is however a fundamental biological feature of malignancies which contributes to uncontrolled tumour growth, eventually leading to death of the host.

Tumour antigens, unlike antigens associated with bacteria and other pathogens, are self-antigens, and the immune system is often tolerant of them. For these reasons, much attention has been given to the development of immunization strategies to maximize the immunostimulatory capacity of dendritic cells (DCs). DCs are a family of professional antigen presenting cells playing a pivotal role in the modulation of T-cell responses; these cells are extremely important in protection from pathogens and in tumour immunology. This realization has boosted fundamental translational research to understand and exploit their unique immunomodulatory capacity against cancer [2].

DC vaccines were shown to be safe, feasible and effective in some patients, particularly if the DCs were appropriately matured and activated [5,6]. Nevertheless, although immunological responses are observed in most instances, clinical responses are only detected in a minority of patients [7]. Several of the early studies published were inadequate in their design and interpretation, as immature rather than mature DCs were used [8]. Opportunities for improving the efficacy of DCs in the immunotherapy of tumours must consider a number of different variables. Thus, recent reports have shown that, compared to immature DCs, mature DCs have a higher potency to induce specific immune responses and to migrate both *in vitro* and *in vivo* [9]. Other characteristics of these cells that need to be considered are their different subsets, the modality of antigen loading, the route of administration, and the dose and frequency of DCs administrations. Finally, the immunizing ability of DCs *in vivo* is critically influenced by their maturation state and their capacity to migrate toward lymphatic tissue.

Large numbers of DCs can be generated by *in vitro* culture of monocytes or CD34^+^ progenitors with granulocyte macrophage-colony stimulating factor (GM-CSF) plus interleukin-4 (IL-4) [10] or IL-13. DCs obtained in this way can be primed with tumour antigens in order to optimize their ability to generate tumour-specific T-cell responses. Thus, cells can be loaded either with whole tumour cells or tumour cell lysate, tumour antigen-enriched fractions, or, alternatively, with tumour-specific antigens. Approaches utilizing whole tumour cells as a source of antigen for DCs may be particularly useful: in this way the entire repertoire of antigens associated with a given tumour can be processed. This could prevent tumour immune escape through antigen-loss variants or mutations in critical T-cell epitopes [11,12]. Tumour cell lysate represents the whole protein content of lysed tumour cells. The advantage of using tumour lysate lies in the fact that the multiple antigens that can sensitize T-cells may be heterogeneously expressed on growing tumours (especially those that do not have molecularly defined TAA). Additionally, the cellular stress induced by lytic processes can elicit adaptive mechanisms, including the expression of heat shock proteins (HSPs), which are released from dead cells after primary or secondary necrosis [13,14]. HSPs may improve recognition and uptake of dying cells by DCs; additionally, tumour-derived antigenic peptides may bind to HSPs and be recycled for antigenic presentation in a particularly efficient manner [14]. Antigen loading is indeed a delicate process as it must not disrupt the expression of MHC class I- and class II- and of co-stimulatory molecules, so to allow DCs to effectively present antigens and prime T lymphocytes. Optimally manipulated DCs must also express a stable as well as an activated phenotype and should be enriched with adhesion molecules and chemokine receptors to allow their homing to secondary lymphoid organs.

The mouse mammary tumour virus (MMTV)-induced human-*Ras* expressing breast tumour animal model is a highly informative model for human breast cancer. [15]. Thus, activating mutations in the Ras oncogene are found in approximately 30% of human malignancies and MMTV-*Ras* mice have been created by placing an activated v-Ha-*Ras* under the control of the MMTV-promoter [16]. Malignant mammary and salivary gland tumours arise among transgenic mice between 9 and 20 weeks with a peak at 12–15 weeks of age. We have previously defined the optimal conditions for labelling whole tumour lysate-loaded DCs for MRI and SPECT imaging [17]. Results of these studies showed that these procedures do not alter DC function and can be used to track the *in vivo* migration of labelled DCs to draining LNs into tumour bearing MMTV-*Ras* transgenic mice.

On the basis of these findings, we further characterized the immunological features of whole tumour lysate-pulsed DCs in terms of cell phenotype, functionality, and ability to elicit specific anti-tumour T-cell responses *in vitro* using the MMTV-*Ras* transgenic mice model. Furthermore, an *in vivo* study was performed by injecting antigen-loaded DCs into the same mouse model, and immune system profile and tumour onset were evaluated.

Data herein reported are able to support DC-mediated activation of the immune system against tumour, showing a semi-mature profile of dendritic cells and a T_H_1 phenotype of T-cells capable of significantly delaying tumour growth.

## Materials and Methods

### Animals

The study was approved by the Italian Ministry of Health (study protocol 07/010 for the animal facility located at the Department of Biomedical and Clinical Sciences “L. Sacco”, Milan). Animals were managed according to the principles of the "Guide for the Care and Use of Laboratory Animals" and in accordance with the Italian national law (Legislative Decree. 116/1992) and the recommendations of the European Community (86/609/CEE) for the care and use of laboratory animals. Adult female FVB mice, 6–8 weeks old, and FVB female MMTV-*Ras* mice [17], 27±8 weeks old, were maintained on a 12-h light-dark cycle in cages of 5 animals with water and food provided *ad libitum*. Male and female MMTV-*Ras* mice bearing tumour lesions were heterozygous for the human-Ras transgene and were maintained in a pure FVB background. MMTV-*Ras* mouse males in the FVB mouse background were bred to wild-type FVB females (Jackson Laboratories) to maintain the FVB background. PCR-based mouse screening assay to identify transgenic mice was performed.

### Dendritic cell culture

Total bone marrow cells were extracted from tibias and femurs of wild-type FVB mice, as previously described [17]. Briefly, after removal of muscle tissue, bone epiphyses were cut and bone marrow was flushed out using a 26G1/2 needle syringe. The cell suspension was cultured in complete ISCOVE (Euroclone, Italy) medium. A specific cytokine cocktail, containing 3000 U/ml GM-CSF and 900 U/ml IL-4 (R&D Systems), was added to the culture medium. On day 3, cells were split 1:2 with fresh complete medium.

### Cell count

Cell count was performed with the automated cell counter ADAM-MC (Digital Bio, NanoEnTek Inc, Korea). ADAM-MC automatic cell counter measures total cell numbers and cell viability by cutting-edge detection technologies. In addition to Trypan blue staining, ADAM-MC procedure was carried out using two sensitive fluorescent dye-staining solutions, AccuStain Solution T (Propidium Iodide/lysis solution) and AccuStain Solution N (Propidium IodideI/PBS). AccuStain Solution T permeabilizes plasma membrane and stains nucleus allowing the measurement of total cell enumeration, while AccuStain Solution N exclusively stains non-viable cells. A 532 nm optic laser is automatically focused onto the cell suspension contained into a disposable microchip where cell analysis is made with a CDD camera.

### Antigen pulsing of DCs

DCs were harvested on day 6, and 1.5x10^6^ cells were seeded in 24-well plates. DC maturation was induced by incubation with whole tumour lysate of mammary tumours explanted from MMTV-*Ras* mice. Tumour cell lysis was performed as previously described [17]. Briefly, mammary tumour lesions from transgenic animal models (MMTV-*Ras*), were mechanically disaggregated, heated at 42°C in a water bath for 1 hour and then for 2 hours at 37°C in CO_2_ 5%. Tumour cells were then digested with 0.02% Trypsin (Euroclone) and washed in PBS (PBI International). Cells were resuspended at 15x10^6^ cells/ml and lysed by 3 cycles of freeze-thawing in liquid nitrogen. The preparation was centrifuged at 12,000 rpm for 15 minutes and stored in aliquots at -80°C until use. Tumour lysates were added to DC culture plates for 24 hours at the ratio of 1 DC to five tumour cell equivalents (i.e.1:5). Non-loaded DCs were used as negative controls and the TLR agonist LPS—Lipopolysaccharide (1 ug/ml) was used as positive control.

### Flow cytometry

0,25x10^6^ DCs were resuspended in PBS and stained with fluorescent labelled monoclonal antibodies directed toward a panel of cell surface markers [fluorescein isothiocyanate (FITC), phycoerythrin (PE), phycoerythrin-cyanin 5 (PCy5) or 7(PCy7)] (eBioscience, San Diego, CA, USA): CD11c, CCR7, MHC-I/II, CD80, CD86, CD40, PD-L1. Following incubation 15 min at room temperature in the dark, cells were washed 3 times in PBS and fixed in 1% paraformaldehyde. Cytometric analyses were performed using an FC500 flow cytometer (Beckman-Coulter, Miami, FL) equipped with a double 15-mW argon ion laser operating at 456 and 488 interfaced with an Intercorp (Venice, Italy) computer. Samples were first run using isotype controls or single fluorochrome-stained preparations for colour compensation. For each analysis 20,000 events were acquired and gated on CD11c expression and side scatter properties. Green fluorescence from FITC (FL1) was collected through a 525-nm band-pass filter, orange-red fluorescence from R-PE (FL2) was collected through a 575-nm band pass filter and red fluorescence from Cy5PE (FL4) was collected through a 670-nm band-pass filter. Data were collected using linear amplifiers for forward and side scatter and logarithmic amplifiers for FL1, FL2, FL4, and FL5.

### Confocal microscopy

Tumour masses were explanted and cells were disaggregated and labelled with PKH26 Red Fluorescent Cell Linker (Sigma Aldrich), according to the manufacturer’s instructions. Labelled cells were lysated, as previously described [17], and DCs were incubated with the labelled lysate for 24 hours. Cells were then harvested and stained with an anti-CD11c FITC monoclonal antibody for 24 hours at 4°C, fixed with paraformaldehyde 1% for 15 minutes, washed and mounted on slides using the Vectashield Mounting Medium (Vector Laboratories, Burlingame, CA, USA). Blue DAPI staining was performed to identify nuclei. Confocal fluorescent images were obtained using a LSM 510 Zeiss confocal scan head mounted on a Zeiss Axiovert 200 M on an inverted-based microscope using a 40x or 63x oil immersion objective. Sequential excitation at 488 nm and 543 nm was provided by argon and helium-neon gas lasers, respectively. Emission filters BP500-550 and LP560 were used for collecting green (FITC) and red (PKH26) in channels one and two, respectively. After sequential excitation, green and red fluorescent images of the same cell were saved with Laser Sharp software. Images were analysed by Zeiss software. The term co-localization refers to the coincidence of green and red fluorescence, as measured by the confocal microscope.

### Real time PCR array

RNA was extracted from DCs, after incubation with tumour antigens for 6, 16 or 24 hours, using the acid guanidium thiocyanate-phenol-chloroform method, dissolved in RNase-free water and purified from genomic DNA with RNase-free DNase (RQ1 DNase, Promega, Madison, Wisconsin, USA). One microgram of RNA was reverse-transcribed into first-strand cDNA in a 20-μl final volume containing 1 μmol/l random hexanucleotide primers, 1 μmol/l oligo dT, and 200 U Moloney murine leukaemia virus reverse transcriptase (Clontech, Palo Alto, California, USA). Real-time PCR experiments were performed with SYBR technology (SYBR Green PCR mix; Finnzymes, Espoo, Finland) using a Dendritic and Antigen Presenting Cell PCR Array (SA Biosciences Corporation, Frederick, Maryland, USA). A 96-well plate containing RT2 qPCR Primer Assays for a set of 84 related genes, focused on dendritic cell activation and maturation, plus five housekeeping genes and three controls. Controls for genomic DNA contamination, RT reaction quality and general PCR performance were included in each array. Significantly upregulated and downregulated genes were only those ones that showed at least a twofold change in the level of mRNA expression of the loaded versus unloaded DCs in three independent experiments (fold change ≥2).

### Naïve syngeneic T-cell harvesting

The stimulatory capacity of antigen loaded DCs was tested by culturing tumour-lysate pulsed DCs with CD3^+^ naïve responder splenocytes collected from MMTV-*Ras* mice after tumour onset. MMTV-*Ras* mice were anesthetized with 4% chloral hydrate v/v (Sigma-Aldrich) and sacrificed by cervical dislocation. Spleens were excised under sterile conditions in a laminar flow hood and put through a 100 mm plastic strainer (BD Falcon 2350, BD Biosciences, Bedford, MA) for cell recovery. Splenocytes were layered on a continuous 40–100% Percoll gradient (Sigma) and washed twice in PBS to obtain lymphocyte-rich cells. Cell viability was determined using Trypan blue staining. Splenocytes were resuspended in complete ISCOVE medium. T-cells were purified using a Pan T Cell Isolation Kit (Myltenyi Biotech, Italy) by depleting magnetically labelled non-T cells by total cells (negative fraction contains CD3^+^ cells). 5x10^5^ cells were then stained with anti-CD3-PECy5 (eBioscences) to assess T lymphocyte purity by flow cytometry. After elution, the resulting cells were > 90% CD3^+^ by FACS analysis.

### Induction of proliferative T-cell responses *in vitro*

3x10^7^ CD3^+^ naïve T lymphocytes were resuspended in 0.1% PBS/BSA (PBI International) at room temperature and stained with 2.5 μg/ml of cell-permeant fluorescein-based carboxy-fluorescein-diacetate-succimidyl-ester (CFSE-DA or CFSE, Sigma Aldrich), which covalently attaches to cytoplasmic components of cells, resulting in uniform bright fluorescence. Upon cell division, the dye is distributed equally between daughter cells, allowing the resolution of cell division by flow cytometry. Cells were incubated at 37°C for 10 minutes, resuspended in 5 ml of complete cold ISCOVE supplemented with 10% BSA (I10 medium) and incubated on ice for 5 minutes. Cells were then washed with I10 medium at 1,200 rpm for 7 minutes, resuspended at a final concentration of 3x10^6^ cells in complete ISCOVE medium and seeded in a 24-well plate (Corning Coster) in the presence/absence of 3x10^5^ tumour-lysate pulsed DCs for 7 days. Cells were then harvested, washed in PBS and stained with anti-mouse CD3-PECy7 (eBioscences) and analysed by flow cytometry. The percentage of proliferating cells was calculated as follows: $\frac{numberofpeak\frac{0}{cells}}{numberofpeak\frac{0}{cells}+numberofeventspeak0}$.

The proliferation index was calculated as follows: $\frac{numberoftotaleventforapeak}{\sum total\frac{events}{replicative}cycles}$.

### Cytokine analysis

10^5^ tumour-lysate pulsed DCs were plated in a 24-well plate with 10^6^ syngeneic CD3^+^ T-cells and incubated at 37°C 5% CO_2_ for 1, 3, 5 or 7 days [5 μg/ml PMA -Phorbol Myristate Acetate- and 1M Ionomycin (Sigma Aldrich)-stimulated T-cells or T-cells alone were used as positive and negative controls, respectively]. Plates were centrifuged at 1,000 rpm for 10 minutes and the supernatants were collected and stored at -20°C for determination of cytokine levels. IFN-γ, IL-12p70 and IL-10 concentration in the supernatants was measured by specific sandwich enzyme-linked immunosorbent assays (ELISA) (R&D System, Minneapolis, MN, USA) according to the manufacturer’s instructions.

For cytokine analysis by flow cytometry, 3x10^5^ tumour-lysate pulsed DCs were plated in a sterile tube with 3x10^6^ syngeneic CD3^+^ T-cells and incubated at 37°C 5% CO_2_ for 1, 3, 5 or 7 days. 10 μg/ml Brefeldin A (Sigma-Aldrich, St. Louis, MO, USA) was added to the cultures during the last 6 hours of stimulation to block protein secretion. Cells were washed in PBS (PBI, Milan, Italy), split in different flow cytometry tubes, and stained for 1 hour at 4°C with an unlabelled antibody anti-FCγR to reduce a-specific signals. Cells were then stained with anti-CD11c PECy5 and anti-CD3-PECy7 (eBioscience) for 30 minutes at 4°C in the dark, washed and fixed in Reagent A solution (FIX & PERM Cell Permeabilization kit; Caltag Laboratories, Burlingame, CA, USA) for 10 minutes at room temperature in the dark. Cells were then washed in PBS and resuspended in reagent B (FIX & PERM Cell Permeabilization kit; Caltag Laboratories, Burlingame, CA, USA) with mAbs specific for different cytokines (IL-12p70, IL-10, IFN-γ, eBioscience). After a 45-minute incubation at 4°C in the dark, cells were washed and fixed in 1% paraformaldehyde in PBS.

### *In vivo* immunization schedule

MMTV-*Ras* mice of 6 weeks of age were randomized in two groups: one group receiving immunization with tumour lysate-loaded dendritic cells, and one group receiving physiological saline as control. For both groups, the injection site at the level of fore pad of left hind limb was pre-treated with 30 ng of TNFα 24 hours before cell administration. Then, 2x10^6^ antigen loaded-DCs were injected in the treated group of MMTV-*Ras* mice, in the presence of further 30 ng of TNF-α [17]. Control mice received TNF-α injection together with 20 ul of physiological saline; untreated mice did not receive any treatments. Mice were treated for two consecutive weeks with the same protocol of injection. Animals were followed weekly to early identify tumour onset by manual palpation and were sacrificed two weeks following tumour establishment by cervical dislocation after anesthetization with 4% chloral hydrate v/v (Sigma-Aldrich). Some mice from each group were sacrificed seven days after receiving the first immunization and spleens were aseptically collected for analysis of T-cell phenotype and measurement of cytokine production; conversely, all remaining mice were followed until the end of the immunization treatment and tumour onset was recorded. Mice then were sacrificed two weeks after tumour establishment and tumour masses were collected.

The onset delay was calculated as: week of onset of treated-mice—week of onset of control mice.

In regards to the analysis of T-cell phenotype, CD3^+^ T-cells were isolated from splenocytes of immunized or control MMTV-*Ras* mice by magnetic separation using a Pan T Cell Isolation Kit (Myltenyi Biotech, Italy) and were stained with fluorescent labelled monoclonal antibodies directed toward a panel of cell surface markers [fluorescein isothiocyanate (FITC), phycoerythrin (PE), phycoerythrin-cyanin 5 (PCy5) or 7(PCy7)] (eBioscience, San Diego, CA, USA): CD4, CD8, CD25, CD69. As a parameter of T-cell effector function, combined evaluation of cytokine secretion was performed by flow cytometry. 3x10^6^ CD3^+^ T-cells were stained for 1 hour at 4°C with an unlabelled antibody anti-FCγR to reduce a-specific signals. Cells were then stained with anti-CD4 PECy5 and anti-CD8-PECy7 (eBioscience) for 30 minutes at 4°C in the dark, washed and fixed in Reagent A solution (FIX & PERM Cell Permeabilization kit; Caltag Laboratories, Burlingame, CA, USA) for 10 minutes at room temperature in the dark. Cells were then washed in PBS and resuspended in reagent B (FIX & PERM Cell Permeabilization kit; Caltag Laboratories, Burlingame, CA, USA) with monoclonal antibodies specific for IL-10, IL-12p70, IFN-γ, TNF-α, Granzyme B and Perforin (eBioscience). A further intracellular staining was performed with a mouse antibody anti-Foxp3 (eBioscience, San Diego, CA) to identify T_regs_ as CD4^+^/CD25^+^/Foxp3^+^ cells. After a 45-minute incubation at 4°C in the dark, cells were washed and fixed in 1% paraformaldehyde in PBS.

### Statistical analysis

Statistical analysis was performed with SPSS 11 software (SPSS Inc., Chigaco, IL, USA). Comparisons of samples to establish the statistical significance of difference were determined by the two-tailed Mann-Whitney rank sum test for independent samples. Values of P < 0.05 were considered significant.

## Results

### DC immunophenotype

**Fig 1:**
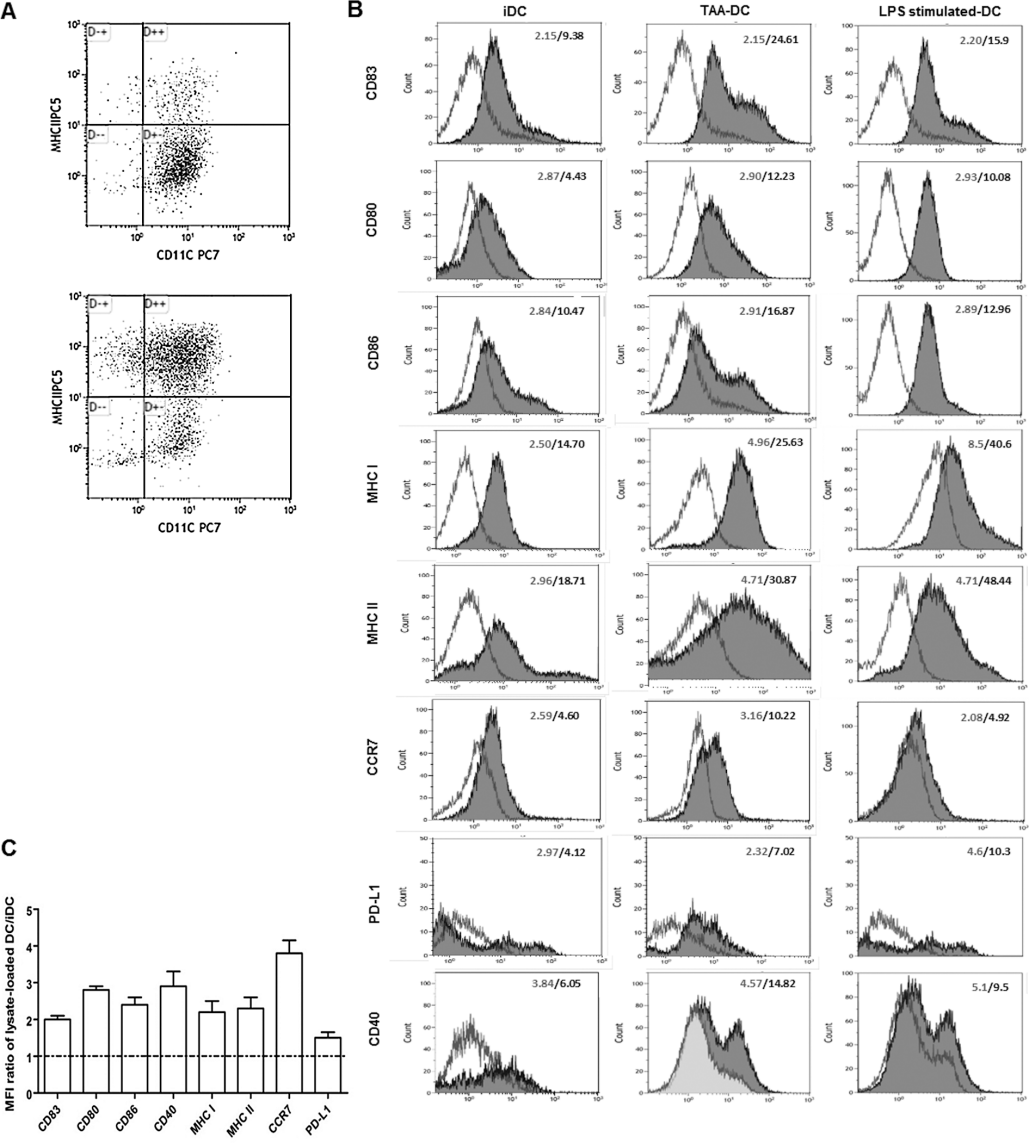
Immunophenotype of day-7 DCs. Unpulsed immature DCs (iDCs), tumour lysate-loaded DCs (TAA-DCs) and LPS stimulated-DCs, were set up in parallel and harvested at the same time for phenotype analysis. DCs were identified by MHC-DR, CD11c (A) (upper dot blot and lower dot blot showing gating strategy and MHC II expression on CD11c^+^ cells before and after tumour lysate-loading and LPS stimulation, respectively) and the markers shown in the figure (B). The numbers indicate the MFI (mean fluorescence intensity) for the isotype controls (open histograms) and DC surface markers (shaded histograms). Representative results from one out of three experiments are shown here. Fold-increase in expression levels of maturation markers on tumour-lysate pulsed DCs over iDCs was determined by expressing the MFIs as a ratio of the TAA-DCs to unpulsed iDCs (C).

Murine DCs were prepared from bone marrow precursors and harvested after 6-day culture in the presence of GM-CSF and IL-4 as described above. The quality and purity of DCs was assessed by FACS analysis on the basis of the expression of the surface marker CD11c; > 88% cells were CD11c^+^. Cell viability was higher than 90% in all experiments. Before loading with tumour lysate, DCs were characterized by flow cytometry to analyse the expression of MHC class I and II molecules, costimulatory molecules (CD86, CD80, CD40), and markers associated with maturation (CD83, CCR7, PD-L1). DCs showed low levels of MHC class I and II molecules, CD86, CD80 and CD40, and CCR7, thus showing an immature phenotype. In all cases DCs expressed significant levels of PD-L1, probably induced by GM-CSF and/or IL-4 treatment (Fig 1).

### DC maturation in response to antigen loading

Tumour cell lysate represents the whole protein content of lysed tumour cells. To induce DC activation and maturation, cells were pulsed with whole tumour lysate of mammary tumours explanted from MMTV-*Ras* mice for 24 hours. As shown in Fig 1, this procedure led to a significant increase in surface expression of specific proteins, including PD-L1, compared with the expression levels of unloaded DCs. Similar results were observed after LPS stimulation (positive control) (Fig 1).

DC viability was not affected by antigen-loading procedure (unloaded-DC: 100% *vs* loaded-DCs and LPS-stimulated-DCs: 98% and 98.5%, respectively), whereas a significant parallel increase in the functional mature DC population could be observed (unloaded-DCs: 11% *vs* loaded-DCs and LPS-stimulated-DCs: 51% and 50%, respectively).

### Confocal microscopy evaluation of DC loading with tumour lysate

**Fig 2:**
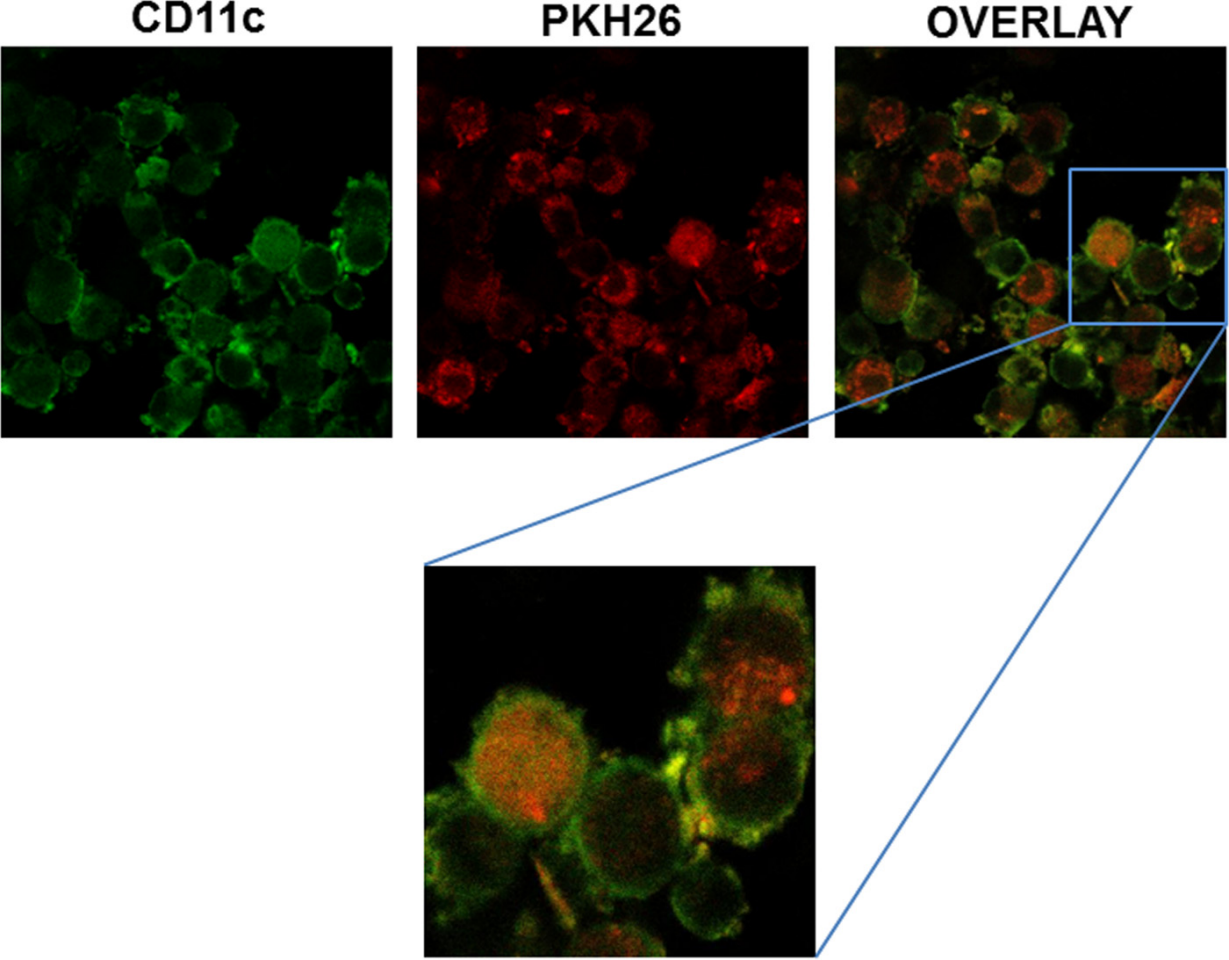
Verification of antigen load by confocal microscopy. Confocal microscopy was used to verify cytosolic localization of tumour-derived antigens following 24 hour co-cultures of day-6 DCs and tumour cell lysate. Tumour masses were labelled with PKH26 Red Fluorescent Cell Linker (red) and DCs were stained with the monoclonal antibody anti-CD11c FITC (green). Antigen load is verified by visualization of tumour antigen (red) within dendritic cells (green). One representative of three independent experiments is shown; all images are 20X whereas zoom is 40X.

The capacity of DCs to take up tumour lysate was confirmed by confocal microscopy. Both murine cells and tumour cell lysate were florescence-labelled and images were acquired. Confocal microscopy pictures of 24 hour co-cultures of DCs and tumour cell lysate show that fluorescent material from lysed tumour cells was internalized by DCs. Analyses of confocal microscopy planes indicated that DC-green fluorescence was surrounding tumour lysate-red fluorescence, but the contrary was not observed. Evidence for internalization in confocal planes is shown at different magnifications and summarized in a reconstruction of multiple confocal planes in the Z axis. Results thus demonstrate cytoplasmic localization of tumour antigens in DCs, confirming that day-6 immature DCs had an active phagocytic ability and were capable to take up whole tumour cell lysate (Fig 2).

### Gene expression profile of tumour lysate-pulsed DCs

To test whether the tumour lysate pulsing modulates the overall gene expression of DCs, we analysed the expression of 84 genes involved in DC activation and maturation using rtPCR arrays. We compared the expression profile of DCs after 6, 16 or 24 hours incubation with tumour antigens; results were confirmed in at least three independent assays.

**Fig 3:**
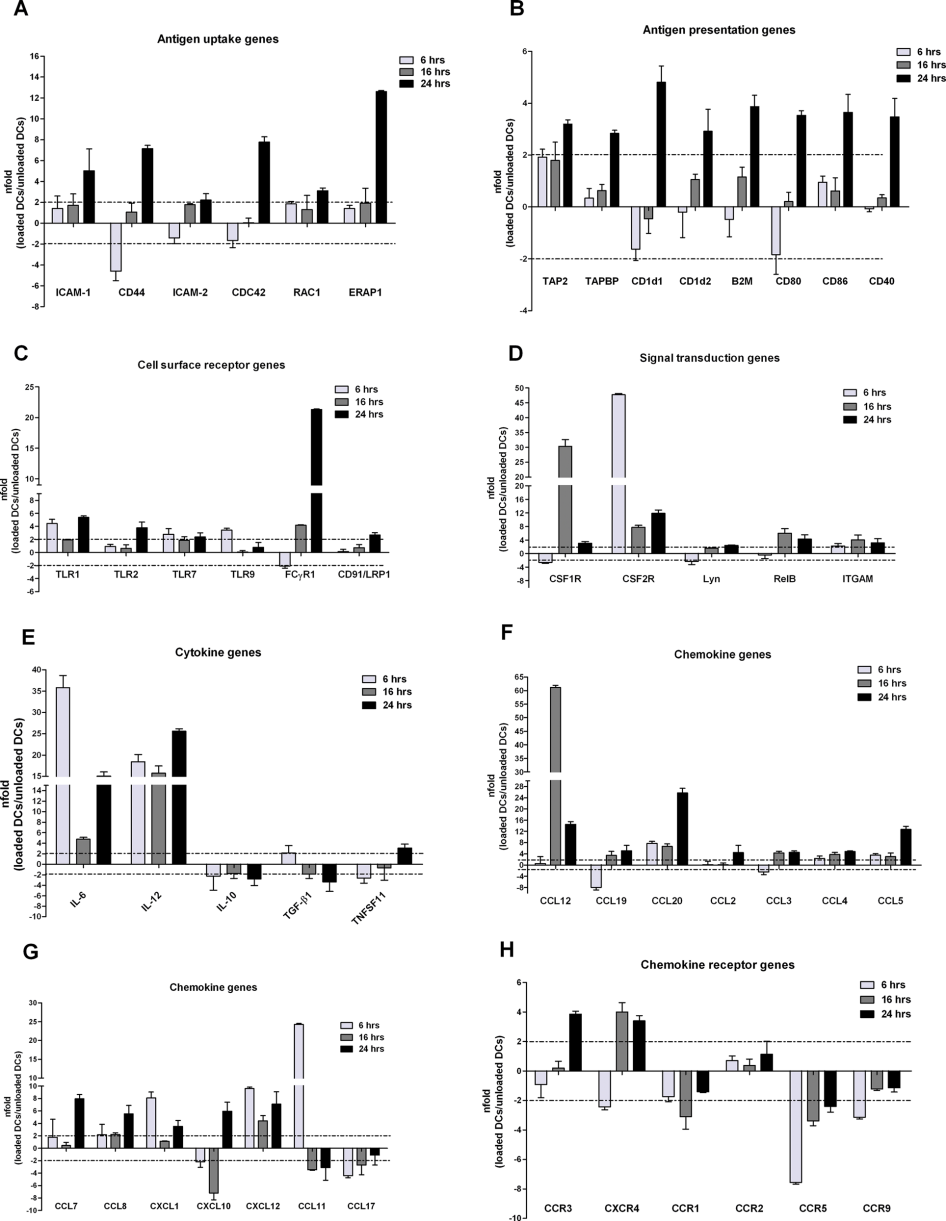
Gene expression profile of tumour-lysate loaded DCs. mRNA expression of genes involved in DC activation and maturation in day-6 DCs following 6-16-24 hour incubation with whole tumour-lysate are shown. The expression of 84 genes involved in antigen uptake/loading (A) and presentation (B), surface receptor signalling (C), signal transduction (D), cytokine (E) and chemokine secretion (F and G) and cytokine and chemokine receptor signalling (H) was assessed by real-time quantitative RT-PCR and shown as fold-change expression from the unloaded DC samples. Controls were also included on each array for genomic DNA contamination, RNA quality, and general PCR performance. Genes showing a twofold change in the level of mRNA expression of the loaded versus unloaded DCs in two of three independent experiments (fold change ≥2) were considered significantly up- or down-regulated. Only the targets showing different expression levels are presented. Mean values ± standard error are shown.

Results showed that 43 genes were upregulated (fold change ≥ 2 in loaded DCs versus unloaded DCs), and 7 genes were downregulated. Among the upregulated genes we found genes that are substantially involved in cell mobility and clustering, and in DC-T cell interactions, such as CD44, ICAM-1, CDC42, RAC1 and ERAP1 (Fig 3A), and in antigen presentation including TAP2, TAPBP, CD1d1 and B2M (Fig 3B). Upregulated genes were also members of the B7 family, including B7.1 (CD80) and B7.2 (CD86), which specifically bind to their cognate ligand on T-cells and provide the second signal for T-cell activation and the enhancement of DC function, and CD40, a costimulatory protein that is required for DC activation by binding to CD40L on T_H_ cells (Fig 3B). Results also showed the upregulation of several surface receptors (Fig 3C), including: 1) members of the Toll-like Receptor (TLR) family, such as TLR1, TLR2 and TLR7, which enhance antigen capture and cross presentation by DCs [18]; 2) FCγR1, which is involved in phagocytosis, antigen processing and cross-presentation via MHC class I molecules and cytokine signalling in adaptive immune responses and in ADCC [19]; and 3) the scavenger receptor CD91/LRP1 (Low-density Receptor-related Protein 1), recently shown to enhance antigen uptake within dendritic cells [20] (Fig 3C). This is consistent with the notion that DCs use specialized immunoreceptors to efficiently internalize antigens before migrating to T cell-rich lymphoid structures. In addition, genes involved in signal transduction were upregulated (Fig 3D), including: 1) CSF1R and CSF2R (GM-CSF), important for DC growth, survival, and differentiation [21]; 2) Lyn, one of the major Src family kinases involved in DC generation and maturation; and 3) the transcription factor NF-κB protein RelB, critical for DC maturation [22]. Finally, cytokines involved in proinflammatory immune response such as IL-6 and IL-12, chemokines and their receptors able to trigger DC migration to lymph nodes (Fig 3E) were positively modulated as well. In particular, results indicated an upregulation of inflammatory chemokines that are produced at low levels in immature DCs [23], such as CCL12, CCL19, CCL20, CCL3 (MIP-1α), CCL4 (MIP-1β), CCL2 (MCP-1), CCL8 (MCP-2), CXCL10, CXCL12 and CCL5 (RANTES) (Fig 3F and 3G), and the receptors CCR3 and CXCR4 (Fig 3H). Notably, CCL3 and CCL4 were expressed rapidly but also transiently, whereas CCL2, CCL8, CXCL10, CXCL12 and RANTES were upregulated at late time-points in a more sustained fashion. On the other hand, genes that characterize the immature state of DCs, such as the inflammatory chemokines CCL11 and CCL17 (Fig 3G) and the chemokines receptors CCR1, CCR5 and CCR9, were down-regulated (Fig 3H) [24]. Notably, IL-10 was downregulated as well. IL-10 is an anti-inflammatory, immunosuppressive cytokine that favours tumour escape from immune surveillance and is involved in the generation of T_reg_ cells with defined immunosuppressive functions (Fig 3E) [25,26]. Finally, TGF-β, which has a role in priming differentiation of IL-10 producing Tr1 cells, was also down-regulated as well in tumour lysate-loaded DCs (Fig 3E).

Taken together these data indicate that pulsing of DCs with whole tumour lysate induces cell maturation and results in a differential modulation of genes involved in DC function and T-cell priming.

### T-cell proliferation induced by antigen-loaded DCs

**Fig 4:**
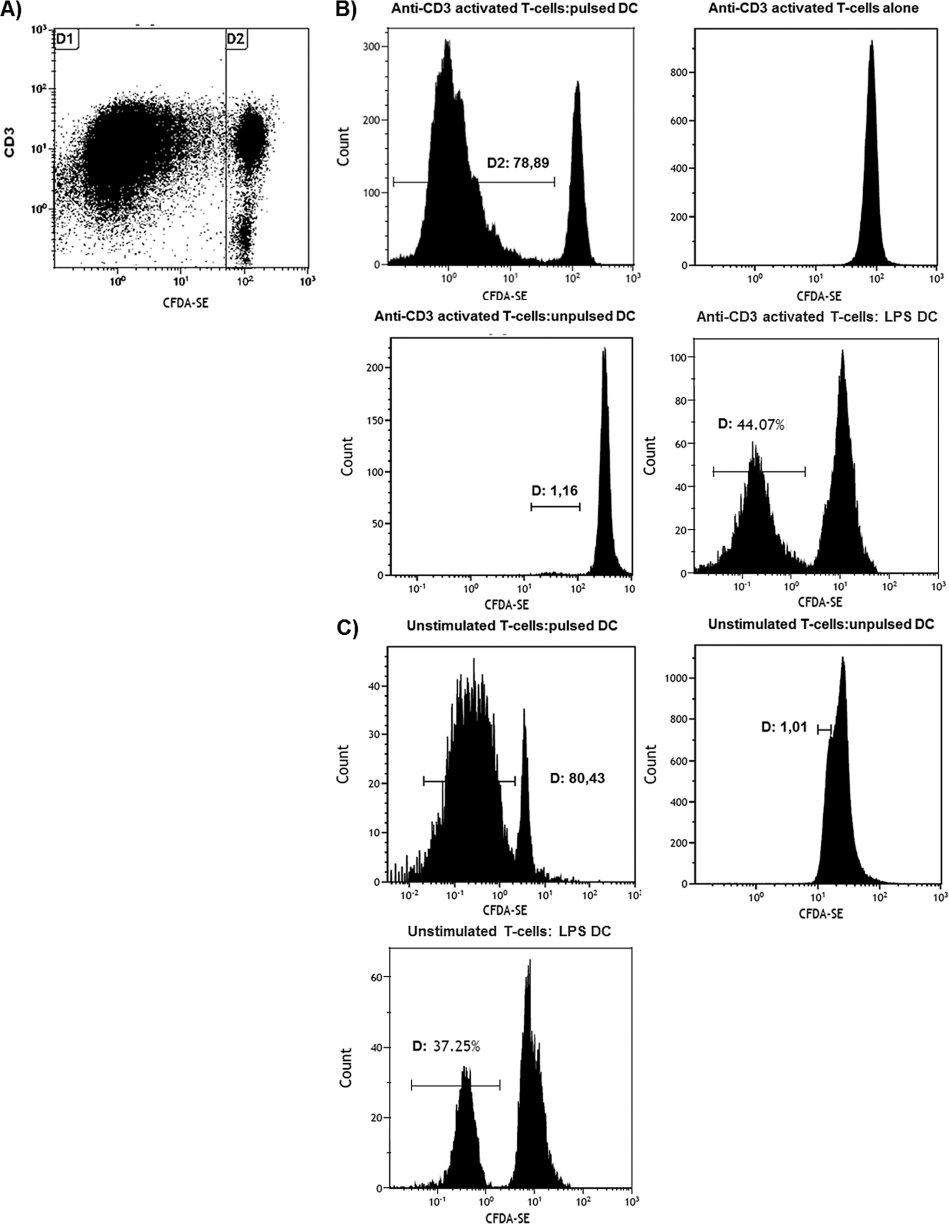
CFSE assay for assessment of T-cell function. The 7 day response of CFSE-labelled syngeneic T-cells from the spleen of MMTV-*Ras* mice to stimulation with tumour lysate-pulsed DCs. CD3/CFSE dot plot (A) and CFSE histogram (B and C, anti-CD3 activated T cells and unstimulated T-cells, respectively) are shown. All plots were gated on CD3-positive cells, while the histograms were also gated to include both resting lymphocytes and blasts. (A): alterations in light scatter characteristics; (B and C): progressive two-fold dilutions of CFSE that accompanied mitotic cell division (gate D1). Both anti-CD3 activated and unstimulated T-cells were incubated with tumour lysate pulsed-DCs, unpulsed DC (negative control) and LPS-stimulated DCs (positive control). Application of an analysis algorithm (see Materials and Methods) resulted in a proliferation index of 2,93. One representative of three independent experiments is shown.

To evaluate the stimulatory properties of tumour lysate-pulsed DCs, we assessed DC-induced T lymphocyte proliferation by quantitative analysis of T-cell division at the single cell level. Syngeneic naїve T-cells from the spleen of MMTV-*Ras* mice were labelled with CFSE, stimulated with anti-CD3, seeded in 24-well tissue culture plates for 7 days in the presence/absence of tumour lysate-pulsed DCs or unloaded control DCs, and analysed by flow cytometry. T lymphocytes stimulated with either PMA and Ionomycine or LPS were used as positive controls. CFSE labelling of T-cells cultured either with antigen-loaded or unloaded DCs 24 h after CD3-stimulation, when division has not started yet, was similar (data not shown). However, the activated state of T lymphocytes cultured with antigen-loaded DCs that was observed at day 7 (see above) was accompanied by cellular division, with 79% T-cells moving from the resting to the blast population (Fig 4) and a proliferation index of 2.93, similar to that of LPS-stimulated DCs (positive control) (Fig 4). No expansion of CFSE^+^ cells could be observed after co-culture with unloaded DCs (negative control). These results show that tumour lysate-pulsed DCs are functionally active *in vitro*, as they are able to elicit the proliferation of T lymphocytes.

### Cytokine production

**Fig 5:**
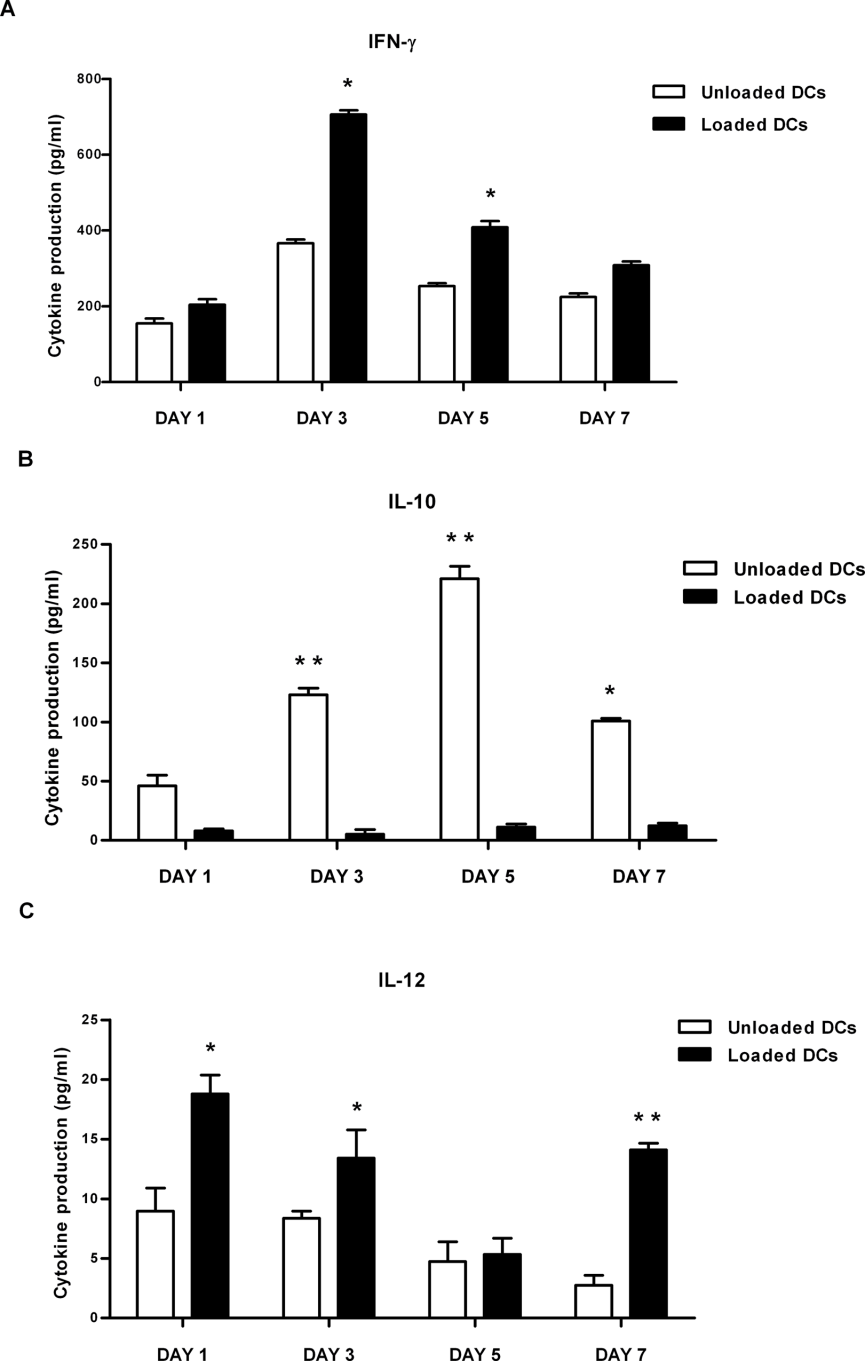
Polarization of CD3^+^ lymphocytes by whole tumour-lysate pulsed DCs. To examine the nature of T-cell responses induced by whole tumour-lysate pulsed DCs, syngeneic naïve T-cells from MMTV-*Ras* mice were cultured with either unloaded DCs or TAA-DCs for 1-3-5-7- days and both T cell- and DC-derived cytokines were measured. ELISA quantified released type 1 [IFN-γ (A), IL-12 (C)] and type 2 [IL-10 (B)] cytokines in the supernatants. Results are expressed as mean values ± standard error of three independent experiments. *P value < 0.05; **P value <0.005.

Type 1 (IFN-γ, IL-12) and type 2 (IL-4, IL-10) cytokines were measured next in supernatants collected from tumour lysate-pulsed DCs co-cultured with syngeneic naїve T-cells. As shown in Fig 5A and 5C, IFN-γ and IL-12 secretion was significantly increased in T-cells co-cultured with tumour lysate-pulsed DCs compared with T-cells co-cultured with unloaded DCs. IL-10 produced in the same conditions was significantly reduced (Fig 5B), whereas IL-4 production was comparable in both conditions (data not shown).

**Fig 6:**
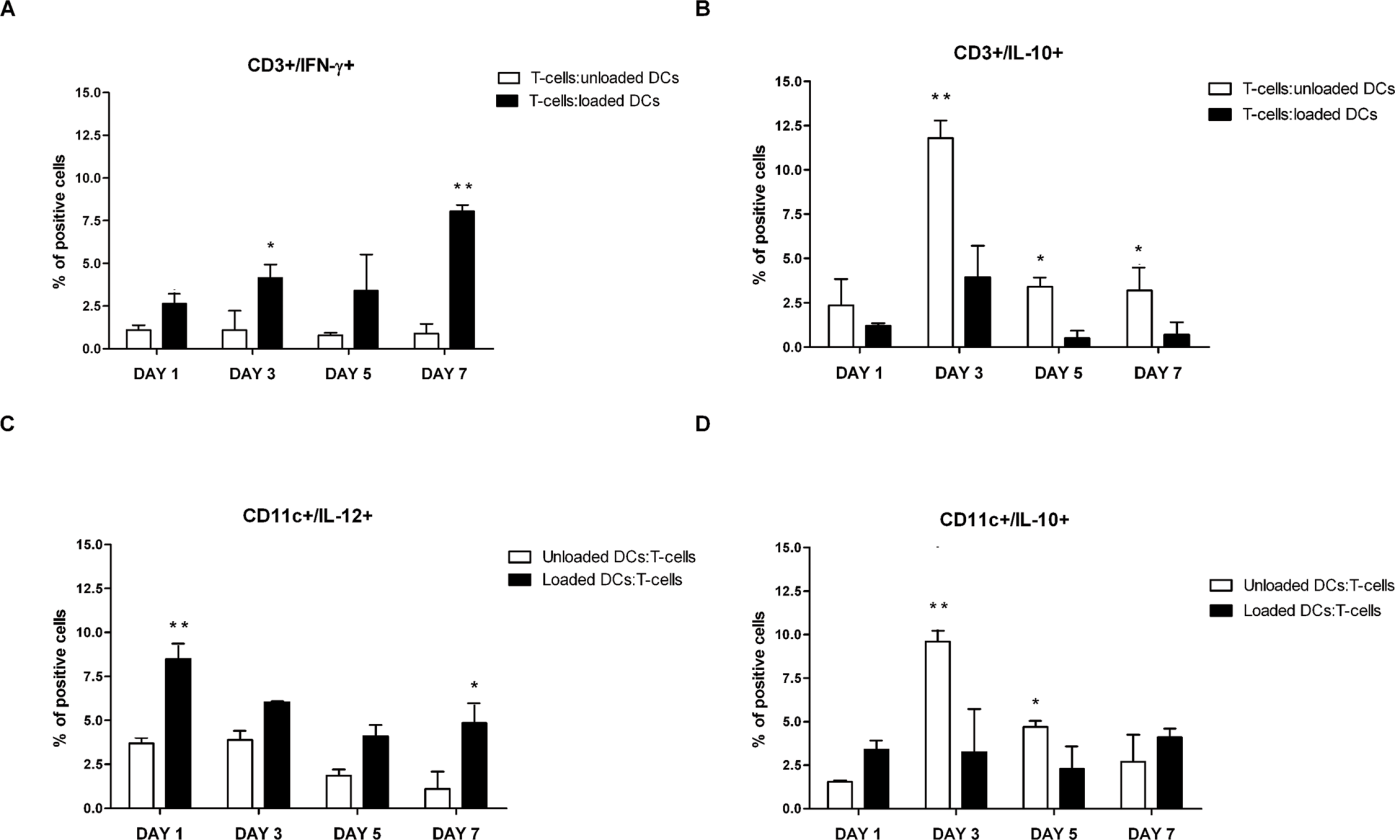
Percentage of IFN-γ- and IL-10-producing CD3^+^ lymphocytes after co-culture with either unloaded or whole tumour-lysate pulsed DCs. Syngeneic naïve T-cells from MMTV-*Ras* mice were cultured with either unloaded DCs or TAA-DCs for 1-3-5-7- days. After the indicated days of DC-T culture, BFA was added to the culture for the last 6 hours before staining to prevent cytokine secretion. Cell were then stained with anti-CD3-PECy7 and anti-CD11c PECy5 mAbs, fixed, permeabilized, stained with PE- or FITC-conjugated mAbs for intracellular cytokines, and subjected to flow cytometry. Percentage of IFN-γ-expressing CD3^+^ lymphocytes (A), IL-10-expressing CD3^+^ lymphocytes (B), IL-12p70-expressing CD11c^+^ cells (C) and IL-10-expressing CD11c^+^ cells (D). Data represents three different lots of independent experiments. Mean values ± SD and statistically significant differences are indicated. For each analysis, 20,000 events were acquired and gated on either CD3 or CD11c expression and side scatter properties. *P value < 0.05; **P value <0.005.

Given that whole tumour lysate potentially contains large number of epitopes for CD4^+^ and CD8^+^ T-lymphocyte priming, we further investigated whether CD3^+^ tumour-specific IFNγ-secreting lymphocytes were being elicited by antigen loaded-DCs by flow cytometry. Results showed that these cells were significantly increased in response to tumour lysate-pulsed DCs compared to T-cells co-cultured with unloaded DCs (Fig 6A). Tumour lysate-pulsed DCs induced the differentiation of T lymphocytes toward the T_H_1 lineage. In particular, as shown in Fig 6B, IL-10-producing T-cells peaked after 3 days of co-culture with tumour lysate-pulsed DCs, prior to the sustained secretion of IFN-γ, but steadily decreased until the end of the culture at day 7. Intracellular staining analysis showed a higher percentage of IL-12-secreting CD11c^+^ cells in tumour lysate-pulsed DC compared to untreated DC culture with T-cells (Fig 6C), that reached statistical significance 1 and 7 days after co-culture. The decrease observed on day 3 and day 5 was consistent with the fairly narrow window of time of IL-12 secretion by DCs.

Similar results were obtained when cytokine production was analysed by ELISA. Notably, since the timing of IL-12 secretion virtually ends 18–24 hours after stimulation, the sustained IL-12 production detected on day 7 could indicate that tumour lysate pulsed DCs generated a second burst of IL-12 secretion. Furthermore, the percentage of IL10-secreting tumour lysate-pulsed DCs was significantly lower compared to untreated DCs after 3 and 5 days of co-culture and slightly increased on day 7 (Fig 6D).

The PD1/PD-L1 interaction suppresses DC maturation and promotes IL-10 production [27]. Interestingly, despite the increase in PD-L1 expression observed in tumour lysate-loaded DCs, IL-10 production was marginal in antigen loaded DCs also when supernatants were analysed by ELISA.

Taken together, these results suggest that DCs pulsed with whole tumour lysate of mammary tumours explanted from MMTV-*Ras* mice display phenotypic maturity and are capable of initiating an adaptive immune response by polarizing T_H_1 immune responses.

### *In vivo* immunization

**Fig 7:**
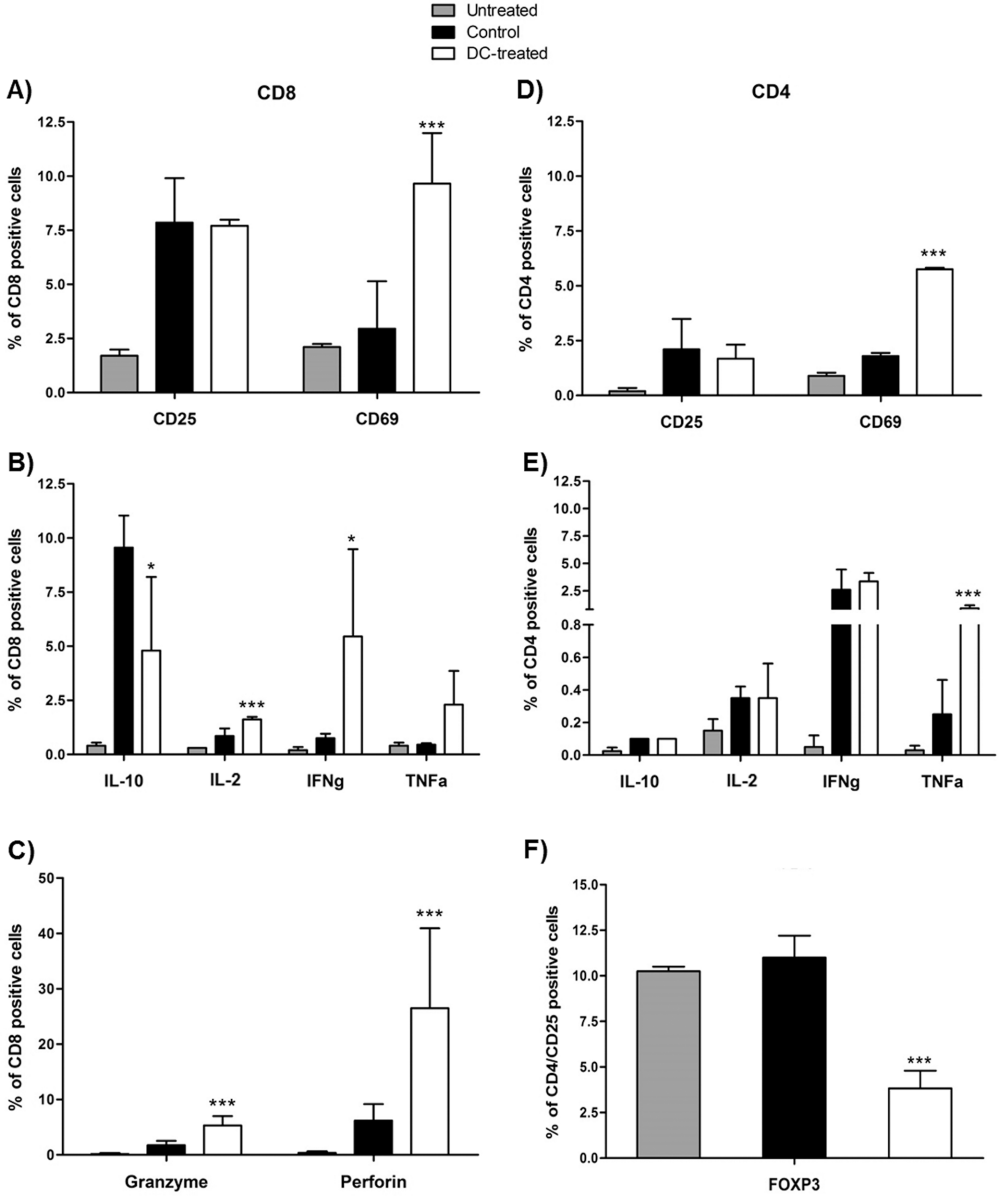
Phenotype of CD4^+^ and CD8^+^ T-cell subsets after *in vivo* immunization. Syngeneic splenocytes from MMTV-*Ras* mice from both untreated, control and DC-treated mice were CD3 enriched by magnetic separation and specifically stained for CD4 and CD8. (A) expression of CD25 and CD69 by CD8 positive cells; (B) production of IL-10, IL-12, IFN-γ and TNF-α by CD8^+^ T-cells; (C) production of Granzyme B and Perforin by CD8^+^ T-cells; (D) expression of CD25 and CD69 by CD4 positive cells; (E) production of IL-10, IL-12, IFNγ and TNF-α by CD4 cells; (F) percentage of CD4^+^/CD25^+^/FOXP3^+^ T-cells. Mean values ± SD and statistically significant differences are indicated. *P value < 0.05; **P value <0.005.

In order to evaluate the effect of antigen-loaded DC dose on subsequent antitumor immune response *in vivo*, MMTV-*Ras* mice were given 2x10^6^ tumour lysate loaded-DCs. Seven days after DC administration, some animals were sacrificed and their spleens dissected for CD3^+^ T-cell isolation and analysis to gather information about the activation status of the immune system. T-cells from DC-treated mice exhibited an overall activated phenotype, characterized by a higher expression of CD25 and CD69 on CD8^+^ T-cells, compared to either control or untreated MMTV-*Ras* mice (Fig 7A). Subsequent measurement of cytokine secretion then represented an important parameter to evaluate CTL effector function. Granule-independent and -dependent CTL effector mechanisms increased after antigen-loaded DC administration. Thus IL-2, TNF-α and IFN-γ production by CD8^+^ T lymphocytes was augmented (Fig 7B), and Perforin- and Granzyme B-expressing CD8^+^ T lymphocytes were increased in DC-treated mice compared to both controls and untreated animals (Fig 7C). Furthermore, a decrease in IL-10 production could be observed in DC-treated mice compared to controls, although the percentage of IL-10 secreting CD8^+^ T-cells remained slightly higher than that observed in untreated mice (Fig 7B).

Similar results were observed in CD4^+^ T-cells. No differences were detected for CD4^+^/CD25^+^ T-cells between DC-treated mice and the control group, but the percentage of the population was still higher compared to untreated mice. On the contrary, the percentage of CD4^+^/CD69^+^ T-cells was higher in DC-treated mice compared to both control and untreated mice (Fig 7D). Results showed a relatively more evident increase in the percentage of CD4^+/^IL-12, CD4^+^/IFN-γ, and CD4^+^/TNF-α secreting T-cells in both DC-treated mice compared to both control and (Fig 7E). Thus, we could observed that the T-cell profile of vaccinated mice was considerably shifted toward the T_H_1 type, supporting the *in vitro* data.

A considerable obstacle to the success of DC-based cancer vaccines might be the presence of T-cells with regulatory function and the potential for DCs to regulate their clonal expansion. In this regard, we verified whether tumour lysate loaded-DCs could facilitate the clonal expansion of T_reg_ cells (CD4^+^CD25^+^ regulatory T-cells) *in vivo*.

Control mice displayed a higher percentage of CD4^+^/CD25^high^/FOXP3 positive cells compared to DC-treated mice (Fig 7F), thus confirming that administration of antigen loaded-DCs does not result in the induction of suppressor cells with a “natural” CD25^+^CD4^+^FOXP3^+^ T_reg_ phenotype.

**Fig 8:**
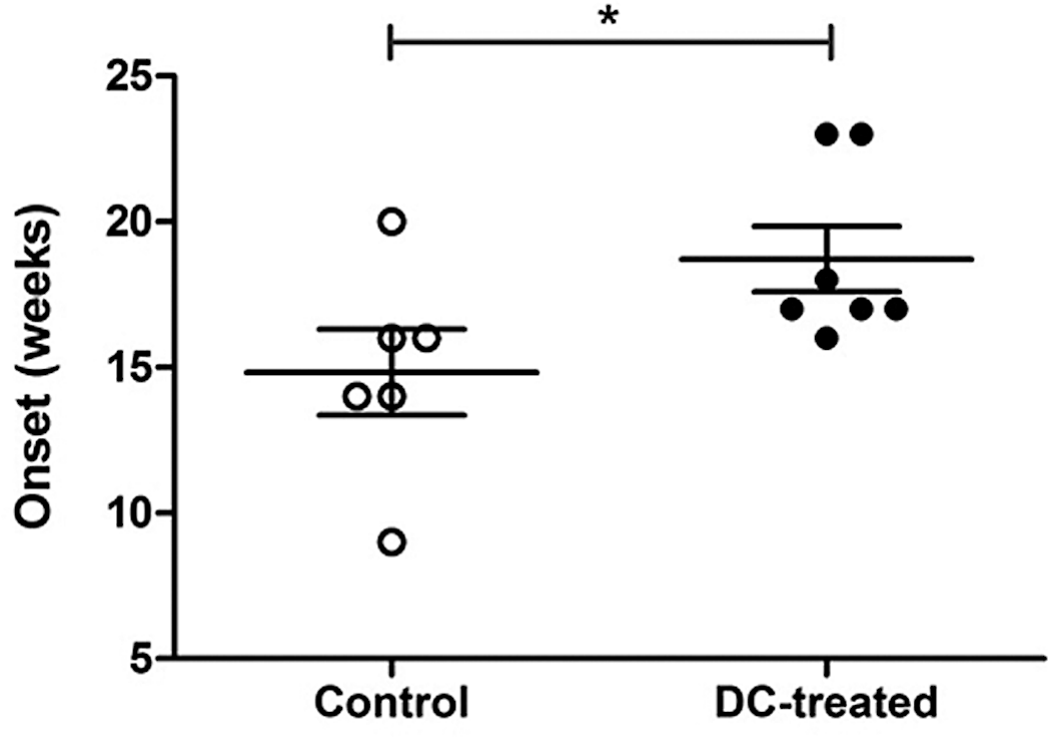
Evaluation of tumor onset. The graph showed the different timing of tumor onset in DC-treated mice versus controls. Mean values ± SD and statistically significant differences are indicated. *P value < 0.05.

In addition, the effect of antigen loaded-DCs on tumour growth inhibition was also assessed. The injection of DC-based vaccine resulted in a significant tumour growth delay of 4 weeks in DC-treated mice compared to control and untreated mice (Fig 8).

## Discussion

DC-based immunotherapies have shown only a limited success in human clinical trials, but recent advances in our understanding of DC biology and increasing evidence that DC vaccines can induce tumour-specific immune responses in cancer patients are leading to renewed optimism for the development of therapeutic DC-based cancer vaccines [27]. We developed a protocol for the *in vivo* evaluation of lymph node homing of tumour-specific DCs in a murine breast cancer model using different imaging techniques [17]. Here we present data from an in-depth functional analyses of bone marrow-derived, cytokine-driven DCs that are pulsed with whole tumour lysate and injected in MMTV-*Ras* transgenic mice. Bone marrow-derived DC were chosen because the yield of CD34-derived was repeatedly shown to be higher and because, even if both CD34- and monocytes derived DCs have comparable phenotype and morphology, the latter are more effective in inducing antigen-specific T-cell responses [28].

DC maturation was induced by incubation with whole tumour lysate of masses extracted from a mammary tumour mouse model (MMTV-*Ras*) [17]. The use of whole tumour lysate with its vast amount of characterized and uncharacterized T-cell epitopes available for activating CD4^+^ T helper and CD8^+^ cytotoxic lymphocytes simultaneously offers distinct advantages in tumour vaccine preparation [29]. Whole protein antigens, DNA, RNA or recombinant viruses encoding the antigen of choice allows host HLA molecules to select the appropriate peptide epitope for presentation on the cell surface; however the spectrum of epitopes recognized by T-cells might be restricted, since certain peptides are not presented by dendritic cells due to missing processing at the level of the proteasome [30]. Ineffective cross-presentation of large protein antigens and low transfection efficiency using cDNA or RNA are further disadvantages to be considered. Although it is easy to synthesize clinical-grade tumor-associated peptides, most of the identified peptides are human leukocyte antigen (HLA)-A2-restricted and patients would be selected as eligible for DC-based therapy only according to their HLA-A2 status. Moreover, the elicited immune responses in cancer patients are restricted to the peptide used for immunization and might be insufficient for controlling tumor growth, since tumor cells frequently undergo high rates of mutation resulting in the loss of a single or multiple antigens. In this regard, whole tumor lysate offers a source of antigens that can elicit a broad polyclonal tumor-specific response directed against multiple antigenic epitopes. The parallel presentation of antigens to both CD4^+^ and CD8^+^ T-cells allows to generate strong primary immune responses to prevent tumour escape. The stimulated CD4^+^ T-cells could also provide help to CD8^+^ T cells to generate long-term memory. In addition, results from a meta-analysis of about 1,800 patients showed that patients who were immunized with whole tumor vaccines had a significantly higher objective response (8.1%) than patients who were immunized with defined tumor antigens (3.6%) [31]. The major disadvantage in using whole tumor in form of lysate is that tumor cells are generally poorly immunogenic. However, there are some protocols to improve the presentation of whole tumour lysate-peptides by MHC class I by induction of Heat Shock Proteins that increase the presentation of exogenous peptides via class I molecules [14]. Furthermore, it has been noted that parameters such as injection route, maturation state and amount of antigen loaded onto administered DCs influence migration of these cells to the lymph nodes, and thus their ability to trigger an effective cytotoxic immune response [32, 33].

To verify that cell loading was achieved, confocal microscopy was used to determine cytosolic localization of antigens following 24-hour co-culture with whole tumour lysate. Results confirmed that efficient antigen transferring resulting in the cytoplasmic localization of tumour antigens was indeed accomplished in DCs. Cell phenotype was evaluated before and after antigen loading, in order to determine differences in DC maturation state. Day-6 culture DCs were in the immature state characterized by low expression of antigen presentation molecules (MHC class I/II molecules), costimulatory molecules (CD80, CD86), maturation markers (CD83, CD40) and of the CCR7 receptor, which is responsible of directing the migration of DCs to the lymph nodes, where they initiate the immune response [34]. Tumour lysate pulsing resulted in a significant increase in the expression of MHC and costimulatory molecules as well as of CD83, a marker of phenotypical maturation [35], CD40 and CCR7. Expression of CD40 was particularly relevant, since signalling through CD40L leads to IL12p70 production and, in turn, to a polarization toward a T_H_1 response. PD-L1 expression was only marginally increased by tumour antigen loading. PD-L1 expression on DCs is consistent with the hypothesis that immature DCs have a balance of stimulatory *vs*. inhibitory molecules that favours inhibition of T-cell responses. Upon maturation, nevertheless, the higher expression of MHC class I/II molecules and B7-1 and B7-2 provides a sufficiently strong stimulatory signal to overwhelm the negative signals delivered via the PD-1/PD-L1 pathway. These considerations, notwithstanding, the observation that PD-L1 expression was only marginally affected by tumour lysate pulsing could help in prevent tumour escape as this molecule plays an active role in the induction and maintenance of T-cell anergy [36].

Transcriptional changes induced by pulsing bone marrow-derived DCs with whole tumour lysate were examined by a pathway-specific APC gene array. Results showed that the majority of the examined genes (49/84) was differentially expressed in DCs upon tumour antigen-loaded, with an up-regulation of those genes encoding proteins involved in antigen uptake and presentation, of molecules for T-cell binding and costimulation, and of TLRs. Furthermore, the expression pattern of signal transduction genes was also significantly increased. A differential regulation of chemokines and cytokines and their cognate receptors was also observed by the gene array analyses. Results showed that the inflammatory chemokine receptors CCR1, CCR5 and CCR9, which guide immature DCs to inflammatory sites where antigen sampling can take place were down-regulated. In contrast, receptors for constitutive chemokines such as CCR3, CCR7 and CXCR4, which drive the maturing DCs towards T-cell areas within the lymph nodes, were upregulated. Upon tumour antigen pulsing, high levels of chemokines that sustain the recruitment of circulating DCs to inflamed tissues, such as CCL3, CCL4, were expressed, whereas at late time-points constitutive lymphoid chemokine such as CCL2, CCL8, CXCL10, CXCL12 and RANTES were selectively up-regulated. IL-6 and IL-12 mRNA expression was similarly up-regulated in whole tumour lysate-pulsed DCs, the production of high levels of IL-12 being characteristic of maturing DCs at the initial state of naïve T cell-DC interaction. These results were further confirmed by the observation that genes characteristic of the immature state of DCs, such as the inflammatory chemokines CCL11 and CCL17 and the cytokines IL-10 and TGF-β were downregulated in pulsed DC. These cytokines are involved in the immunosoppression of tumour-specific immune responses and are associated with the induction of IL-10-producing T_reg_ cells in response to tumours [37].

Bone marrow derived, whole tumour lysate-pulsed DCs were efficient activators of T lymphocytes proliferation and T_H_1-type immune responses *in vitro*. Furthermore, tumour-specific T-cells primed with these DCs released large amounts of cytokines such as IFN-γ, whose biological activity is associated with cytostatic/cytotoxic and antitumour mechanisms, and low levels of IL-10, which favour tumour growth [38,39]. In T lymphocyte/pulsed DC co-cultures, T-cell production of IFN-γ was likely supported by IL-12p70, a potent inducer of T_H_1 polarization, whose secretion was high on day 1 and 7 of co-culture; both cytokines have been shown to have a variety of both direct and indirect effects on tumours and to be critical for tumour rejection in a number of models [40,41,42]. This was not the case for unloaded DCs, where secretion of IL-12p70 was markedly reduced. Notably, the second burst of IL-12 secretion observed on day 7 could be due to a re-stimulation by CD40L-expressing T-cells, although this observation should be further confirmed. IL-10 production was significantly reduced, but never totally suppressed in tumour lysate-pulsed DCs. This could be explained by the observation that dendritic cells that are grown and matured *in vitro* synthesize IL-10 in a continuous manner [43].

We also assessed the impact of tumour lysate loaded-DCs on host immunocompetent cell activity *in vivo*. In this regard, MMTV-*Ras* mice were vaccinated with the BM-DC-based vaccine and some of the animals were sacrificed seven days after DC injection for a preliminary analysis of the T-cell compartment, whereas all remaining animals were followed up until the end of the immunization procedures to evaluate tumour growth inhibition. CD3^+^ T-cells were isolated from primed *in vivo* splenocytes and their cytotoxic activity as well as their ability to produce T_H_1-type cytokines were analysed. DC-treated mice produced a high amount of IFN-γ and TNF-α by both CD4^+^ and CD8^+^ T-cells compared to control and untreated mice. IL-10 production was also observed, but was significantly lower in vaccinated animals compared to the control group. Furthermore, CD8^+^ T-cells showed increased cytolitic activity in terms of either Granzyme B or Perforin production in DC-treated mice compared to both control and untreated animals. At the same time, DC-treated mice showed a lower percentage of regulatory CD4^+^ T cells compared to controls, confirming again the strong activation of the immune system toward a T_H_1 phenotype. Most importantly, administration of tumour lysate loaded-DCs in the MMTV-*Ras* mouse model resulted in a tumour delay of 4 weeks, thus demonstrating that the DC-based vaccine may activate host immune system and cytotoxicity mechanisms that play an important role in anti-tumour response.

In conclusion, data herein indicate that whole tumour lysate- pulsed DC indeed acquire a semimature/mature state, which likely results in their ability to induce a tumour-specific response *in vitro* and *in vivo*; these results could be useful in designing DC-based efficient immunotherapies. Our study confirms that full evaluation of bone marrow derived-DC maturity stage after pulsing with whole tumour lysate from MMTV-*Ras* mice provides enough information on their *in vitro* and *in vivo* functionality for further refining the strategy to develop potent, highly immunogenic tumour-based DC vaccines.

## Conclusions

DC loaded with MMTV-*Ras* whole tumour lysates acquire a semimature/mature phenotype characterized by the expression of costimulatory molecules and a T_H_1 profile; this approach should be investigated in the design of anti-neoplastic vaccination protocols against breast cancer.

## Abbreviations

DC: dendritic cells
iDC: immature dendritic cells
TAA: tumour-associated antigen
MMTV: murine mammary tumor virus
Ras: human-Rat sarcoma
GM-CSF: granulocyte macrophage-colony stimulating factor
HSP: heat shock proteins
MRI: magnetic resonance imaging
SPECT: single photon emission computed tomography
BFA: brefeldin A
PMA: phorbol myristate acetate
MFI: mean fluorescence intensity
ICAM-1: intercellular adhesion molecule 1
Rac1: Ras-related C3 botulinum toxin substrate 1
ERAP1: endoplasmic reticulum aminopeptidase 1
TAP2: Antigen peptide transporter 2
TAPBP: TAP-associated glycoprotein or tapasin
LRP1: low-density receptor-related protein 1
CFS1R: colony stimulating factor 1 receptor
CFS2R: colony stimulating factor 2 receptor
Tr1: type 1 regulatory T cells
PD1: programmed cell death protein 1 receptor
PDL1: programmed death-ligand 1
T_reg_: T regulatory cells
