# Correction: Immunological Characterization of Whole Tumour Lysate-Loaded Dendritic Cells for Cancer Immunotherapy

**DOI:** 10.1371/journal.pone.0151008

**Published:** 2016-03-02

**Authors:** Veronica Rainone, Cristina Martelli, Luisa Ottobrini, Mara Biasin, Gemma Texido, Anna Degrassi, Manuela Borelli, Giovanni Lucignani, Daria Trabattoni, Mario Clerici

Dr. Gemma Texido should be included in the author byline. She should be listed as the fifth author, and her affiliation is #8: BU Oncology, Nerviano Medical Sciences, viale Pasteur 10, 20014 Nerviano, Milan, Italy. The contributions of this author are as follows: Conceived and designed the experiments, contributed reagents/materials/analysis tools, and specifically performed the experiments.

Dr. Anna Degrassi should be included in the author byline. She should be listed as the sixth author, and her affiliation is #8: BU Oncology, Nerviano Medical Sciences, viale Pasteur 10, 20014 Nerviano, Milan, Italy. The contributions of this author are as follows: Conceived and designed the experiments, contributed reagents/materials/analysis tools, and specifically performed the experiments.

The correct citation is: Rainone V, Martelli C, Ottobrini L, Biasin M, Texido G, Degrassi A, Borelli M, Lucignani G, et al. (2016) Immunological Characterization of Whole Tumour Lysate-Loaded Dendritic Cells for Cancer Immunotherapy. PLoS ONE 11(1): e0146622. doi:10.1371/journal.pone.0146622
